# Antibiotic Susceptibility and Molecular Typing of Invasive *Haemophilus influenzae* Isolates, with Emergence of Ciprofloxacin Resistance, 2017–2021, Italy

**DOI:** 10.3390/microorganisms11020315

**Published:** 2023-01-26

**Authors:** Maria Giufrè, Rita Cardines, Manuela Marra, Maria Carollo, Marina Cerquetti, Paola Stefanelli

**Affiliations:** 1 Department of Infectious Diseases, Istituto Superiore di Sanità, 00161 Rome, Italy; 2 Core Facilities Technical-Scientific Service (FAST), Istituto Superiore di Sanità, 00161 Rome, Italy

**Keywords:** *Haemophilus influenzae*, surveillance, MLST, antibiotic resistance, whole genome sequencing, quinolone resistance

## Abstract

*Haemophilus influenzae* invasive disease is a severe infection that needs rapid antibiotic therapy. The aim of the study was to perform and evaluate the serotype distribution, antibiotic susceptibility and molecular characteristics of 392 *H. influenzae* invasive isolates collected during 2017–2021 in Italy. The majority of isolates were NTHi (305/392, 77.8%), followed by Hib (49/392, 12.5%). Ampicillin resistance was frequently detected (85/392, 21.7%): 12.2% were β-lactamase producers (all *bla*_TEM_ except one *bla*_ROB_), 9.4% were β-lactamase-negative ampicillin-resistant (BLNAR), with mutations in the *fts*I gene. Six isolates were resistant to ciprofloxacin, with substitutions in GyrA and ParC. An MLST analysis revealed the occurrence of international resistant clones, such as ST103 and ST14, highlighting the importance of molecular surveillance.

## 1. Introduction

*Haemophilus influenzae* (*H. influenzae*) is a Gram-negative microorganism, commonly colonizing the upper respiratory tract in humans, especially children, that can act as a reservoir for this pathogen [1,2] and can cause various infectious diseases, from respiratory tract infection to invasive disease [3]. *H. influenzae* isolates are classified, based on their capsular status, as encapsulated (type a through f) and non-encapsulated or non-typeable (NTHi). Vaccination against *H. influenzae* type b (Hib) in recent decades has led to a great reduction in Hib invasive disease in countries that adopted this vaccination in their immunization program [4]. Nevertheless, in recent years, an increase in the number of cases, mainly due to non-typeable strains that are non-vaccine preventable, has been observed [5,6]. Prompt antibiotic treatment is crucial in the management of *H. influenzae* invasive disease, and β-lactams are commonly used to treat *H. influenzae* infections, but resistant isolates are frequently found with the first report of resistance to ampicillin in the 1970s [7]. Isolates resistant to β-lactams can produce β-lactamase or possess substitutions in penicillin-binding protein (PBP3, encoded by the *fts*I gene), leading to resistance to penicillins and eventually to cephalosporins in β-lactamase-negative ampicillin-resistant isolates (BLNAR) [8]. Moreover, isolates with both mechanisms (the production of β-lactamase and altered PBP3 with reduced affinity to β-lactams) which are resistant to amoxicillin/clavulanate (BLPACR) are now emerging [8]. Other antibiotics used to treat *H. influenzae* infections are quinolones and carbapenems for serious infections. The emergence of resistance to quinolones among *H. influenzae* isolates has been reported in recent years, mainly from Japan [9]. For these reasons, concern regarding the emergence of NTHi and antibiotic resistance placed *H. influenzae* on the priority list by the WHO for research on new antibiotics [10].

In Italy, invasive *H. influenzae* isolates are collected through the National Surveillance of Invasive Bacterial Diseases (Circolare 9 maggio 2017 «Prevenzione e controllo delle malattie batteriche invasive prevenibili con vaccinazione». https://www.trovanorme.salute.gov.it/norme/renderNormsanPdf?anno=2017&codLeg=59229&parte=1%20&serie=null, accessed on 27 November 2022).

In the present study, we analyzed the serotype distribution, antibiotic susceptibility and molecular characteristics of *H. influenzae* invasive isolates to investigate resistance trends and detect emerging resistant clones. β-lactam-resistant isolates were characterized by the determination of penicillin binding protein 3 (PBP3) substitutions, *fts*I allele and multi locus sequence typing (MLST).

## 2. Materials and Methods

### 2.1. Bacterial Strain Collection and PCR Capsular Genotyping

Confirmed *H. influenzae* isolates from invasive disease cases by peripheral laboratories were collected in the period January 2017–December 2021. A case of invasive *H. influenzae* disease was defined according to the European Union case definition as the isolation of *H. influenzae* and/or detection of its nucleic acid from a normally sterile site [11]. Isolates were sent to the National Institute of Health, Istituto Superiore di Sanità (ISS), Rome, in the framework of the National Surveillance of Invasive Bacterial Disease.

The capsular genotype of each isolate was identified using PCR, following procedures previously reported, with PCR amplification of the *omp*P2 and *bex*A genes to confirm the *H. influenzae* species and analyze the capsulation status, respectively [12,13].

### 2.2. Antibiotic Susceptibility Testing and Characterization of Resistance Genes to β-Lactams

For each isolate, the minimum inhibitory concentrations (MICs) of ampicillin, amoxicillin-clavulanate, cefotaxime, ciprofloxacin and meropenem were determined using MIC test strip (Liofilchem S.r.l., Roseto degli Abruzzi, Italy) with Mueller–Hinton agar with 5% defibrinated horse blood and 20 mg/l NAD (MHF) agar plates (bioMérieux, Lyon, France). The interpretative breakpoints were based on the European Committee on Antimicrobial Susceptibility Testing (EUCAST) criteria v12.0 (http://www.eucast.org/clinicalbreakpoints/ accessed on 15 October 2022) [14]. β-lactamase production was revealed using the nitrocefin test (Liofilchem S.r.l., Roseto degli Abruzzi, Italy). *H. influenzae* ATCC49766 was used as control. Multi-drug resistance (MDR) was defined as resistance to at least three different antibiotic classes.

The presence of the *bla*_TEM_ or *bla*_ROB_ genes was investigated in the β-lactamase-producing ampicillin-resistant (BLPAR) isolates using PCR as previously described [15]. In BLNAR isolates, modifications in the PBP3 were studied by sequencing the *ftsI* gene from positions 1197620 to 1199884 of the *H. influenzae* strain Rd KW20 sequence (GenBank accession No. NC_000907) [16]. *fts*I allele was determined by analyzing a 621-bp gene fragment corresponding to nucleotides 977–1597 of the *fts*I gene coding for PBP3 protein through the PubMLST database [17]. This region of *fts*I encodes the region between the amino acid 326 and 533 that includes the three functional motifs of the PBP3, STVK, SSN, and KTG that start at positions 327, 379 and 512, respectively [17].

The deduced amino acid sequences of the transpeptidase domain of PBP3 (amino acids 311 to 570) were aligned in comparison with the corresponding sequence from *H. influenzae* strain Rd KW20 (GenBank accession No. AAC22787.1). Isolates were grouped based on PBP3 amino acid substitutions using the Ubukata et al. and Dabernat et al. criteria [18,19,20]. Briefly, the substitutions D350N, A502T and N526K in PBP3 are known to be associated with ampicillin resistance. Group I is characterized by the presence of the substitution R517H, group II by N526K and group III by M377I and S385T [20]. Group II is further divided into four subgroups, depending on the presence of additional substitutions (group IIa to IId) [20]. Some combinations of substitutions in PBP3 are not included in any specific group (m, miscellaneous). The substitutions M377I, S385T and L389F are associated with resistance to cephalosporins [8].

The quinolone resistance determining region (QRDR) of the *gyrA* and *par*C (encoding DNA gyrase and DNA topoisomerase IV subunit A, respectively) genes was examined in the ciprofloxacin-resistant isolates according to the methods previously reported [21]. Amino acid changes were identified in the QRDRs of GyrA and ParC for each isolate by comparison of the corresponding translated proteins with the quinolone-susceptible reference *H. influenzae* Rd KW20 sequences (GenBank accession No. AAC22917.1 and AAC23175.1, respectively), using protein blast at NCBI. Ciprofloxacin-resistant isolates were defined based on changes in the GyrA protein at positions Ser-84 and/or Asp-88 and in ParC protein at position Ser-84.

### 2.3. MLST, WGS and Phylogenetic Analysis

All the isolates resistant to at least one of the five antibiotics tested along with all the encapsulated isolates were genotyped using MLST following procedures described online, according to the *H. influenzae* MLST website scheme (https://pubmlst.org/hinfluenzae/ accessed on 29 September 2022), by sequencing 450-bp internal fragments of 7 housekeeping genes (*adk*, *atp*G, *frd*B, *fuc*K, *mdh*, *pgi* and *rec*A). Clonal complexes (CC) were defined as clusters of different STs sharing at least five of seven identical alleles and were assigned on MLST website along with STs.

Based on MLST combination of alleles, a minimum spanning tree was generated by applying PHYLOViZ Online (https://online.phyloviz.net/index accessed on 29 September 2022), that uses the goeBURST algorithm to assess and visualize the genetic relationships amongst STs [22].

All the isolates resistant to ciprofloxacin were submitted to whole-genome sequencing (WGS). Genomic DNA was extracted from an overnight culture using the NucleoSpin DNA extract kit (Macherey-Nagel, Duren, Germany). Sequencing was obtained using Ion Torrent (Life Technologies, Thermo Fisher Scientific, Waltham, MA, USA) technologies, according to the manufacturer’s instructions. De novo assembly of sequence reads was performed using SPAdes v3.14 software through the ARIES public Galaxy server (https://w3.iss.it/site/aries/ accessed on 27 November 2022). In silico resistome analysis of the assembled contigs was performed using tools available at the Center for Genomic Epidemiology (CGE) for resistance gene content using ResFinder tool (https://cge.cbs.dtu.dk/services/ accessed on 27 November 2022).

## 3. Results

### 3.1. Serotyping

A total of 392 *H. influenzae* invasive isolates were collected in the 5 years period. Cultured isolates were recovered from CSF (69 isolates) and blood (322 isolates) (missing information for one isolate). The serotype distribution of *H. influenzae* isolates highlighted that the majority were identified as NTHi (305/392, 77.8%), followed by Hib (49/392, 12.5%), Hif (31/392, 7.9%), Hia (4/392, 1%) and Hie (3/392, 0.8%).

### 3.2. Antibiotic Susceptibility Testing

Table 1 shows the antibiotic susceptibility testing results of the 392 *H. influenzae* isolates to the five antibiotics tested. Ampicillin resistance was the most frequently detected: 85 ampicillin-resistant isolates (85/392, 21.7%) were identified, with an MIC range of 1.5–≥256 mg/L. Forty-eight isolates (48/392, 12.2%) were β-lactamase producers; the remaining 37 (37/392, 9.4%) were β-lactamase-negative ampicillin-resistant (BLNAR). The rate of ampicillin resistance varied by serotype: ampicillin-resistant isolates were mainly found among NTHi isolates (26.6%, 81/305) and in a few encapsulated isolates (three Hib isolates and one Hie isolate). Seventeen isolates were resistant to amoxicillin/clavulanate (17/392, 4.3%, MIC range 3-12 mg/L): two were β-lactamase-positive amoxicillin/clavulanate-resistant (BLPACR), 13 were BLNAR, and two were amoxicillin /clavulanate-resistant but susceptible to ampicillin (AMCR). Four isolates were resistant to cefotaxime (4/392, 1.0%, MIC range 0.19–1.5 mg/L); three were BLNAR, and one produced β-lactamase. All isolates were susceptible to meropenem (MIC_50_ = 0.064 mg/L; MIC_90_ = 0.19 mg/L). Six isolates were resistant to ciprofloxacin (6/392, 1.5%, MIC range 0.125–32 mg/L); all were NTHi except one Hif isolate.

Combined resistance was detected in 17 isolates: 12 were resistant to ampicillin and amoxicillin/clavulanate, 2 were resistant to ampicillin and cefotaxime, 2 were resistant to ampicillin, amoxicillin/clavulanate and cefotaxime, and one was resistant to ampicillin, amoxicillin/clavulanate and ciprofloxacin.

A *bla*_TEM_ gene was identified by PCR in all β-lactamase-producing isolates, except one isolate harboring *bla*_ROB_. *fts*I alleles were investigated in 41 isolates (37 BLNAR, 2 BLPACR and 2 two isolates resistant to amoxicillin/clavulanate but susceptible to ampicillin) (Table 2). Overall, we found 23 different alleles: *fts*I allele 1 was the most frequently observed (n = 9), followed by allele 2 (n = 6). The analysis of the deduced amino acid sequences of the transpeptidase region of PBP3 from all these 41 isolates showed the presence of several amino acid substitutions (Table 2). The majority of BLNAR isolates (19/37, 51.4%) were classified into group IIb, defined by the presence of the A502V and N526K substitutions along with other substitutions. The remaining isolates were distributed among group IIc (six isolates, characterized by the presence of the A502T and N526K substitutions), group III-like (three isolates, with M377I, S385T and R517H), group I (two isolates), group IIa (with N526K substitution alone) and IId (with N526K and I449V) (one isolate each), while five isolates were not included in any group. The four isolates that were resistant to cefotaxime belonged to group IIb and III-like, respectively. These isolates exhibited amino acid substitutions at the SSN motif (D350N and M377I) with other substitutions that were variably present including S357N, S385T and N517H. Two isolates resistant to amoxicillin/clavulanate but susceptible to ampicillin belonged to group III-like in one case and to no group in the other.

All six isolates resistant to ciprofloxacin presented different amino acid substitutions in the quinolone resistance determining region (QRDR) (Table 3). Two isolates with low ciprofloxacin MIC (0.125 to 0.5 mg/l) had one substitution only in GyrA (D88N) or one in GyrA (S84L) and one in ParC (S84R), respectively. All the isolates with high resistance to ciprofloxacin up to 32 mg/l exhibited double substitutions in GyrA at amino acid Ser84 and Asp88 and one substitution in ParC at amino acid Ser84. In GyrA, the Ser84Leu amino acid change was detected in each of the four strains; conversely, a different substitution took place at the Asp88 amino acid, with the Asp88Gly or Asn88Tyr amino acid changes. In ParC, the additional single substitution Ser84Ile was detected. The *in silico* resistome analyses revealed that all but one isolate had no mutations in the *fts*I gene and were negative for resistance genes towards aminoglycosides, β-lactams, sulphonamides, trimethoprim, chloramphenicol, fosfomycin and tetracycline. One isolate was MDR and exhibited resistance to ampicillin, amoxicillin/clavulanate and ciprofloxacin. This isolate harbored the genes *bla*_TEM-1B_, *msr*(D) for macrolide, lincosamide and streptogramin B resistance (MLS) and *mef*(A) for macrolide resistance, in addition to mutations in the *fts*I gene (allele 43, substitutions in PBP3: D350N, G490E and N526K) (Table 2).

### 3.3. Molecular Typing

**Figure 1:**
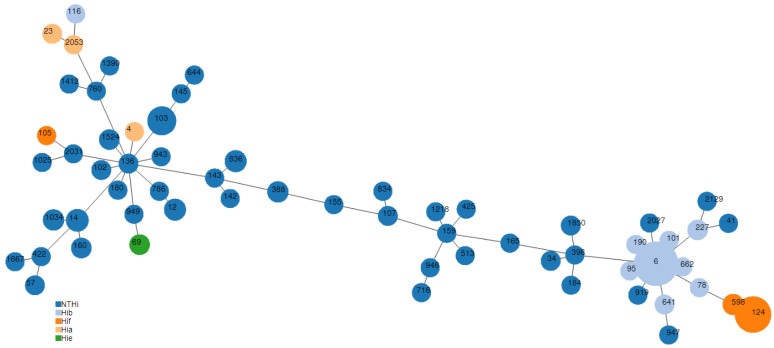
Minimum spanning tree based on MLST alleles showing genetic relationships among STs of 176 *H. influenzae* isolates from invasive disease, Italy, 2017–2021. Each node represents a different ST with the size of nodes proportional to the number of included isolates. ST number is indicated in each node. Capsular type is indicated in different colors, as shown in the figure.

An MLST analysis was performed on all the isolates resistant to at least one antibiotic and on all encapsulated isolates. Of the 175 isolates characterized by MLST, a total of 61 different sequence types were detected, as shown in the minimum spanning tree in Figure 1 and in Supplementary Table S1. 

The 87 encapsulated isolates were clonal and distributed in 16 STs, according to the capsular type: Hib isolates belonged mainly to ST6 (38/49), Hif to ST124 (26/31), Hia to ST23 (2/4) and Hie to ST69 (3/3). The largest clonal complexes associated with NTHi included CC3 (20/88) and CC11 (18/88). Hib isolates were included almost exclusively in CC6, with Hif in CC124 (Supplementary Table S1). 

Forty-eight β-lactamase producers belonged to 23 different STs, with ST103, ST388 and ST836 the most frequently detected (15/48, 31.3% and 4/48 isolates, 8.3%, respectively) (Table 4). The 37 BLNAR isolates belonged to 23 different STs, with ST14, ST1034 (a single locus variant of ST14) and ST12 the most frequent (6/37, 16.2%; 4/37, 10.8%; and 5/37, 13.5%, respectively) (Table 4). ST14 was associated with *fts*I allele 1 and BLNAR group IIb in almost all cases (5/6), while ST12 was associated with allele 2 and BLNAR group IIb in half of the cases (Table 2). No regional outbreaks were detected.

Isolates resistant to cefotaxime belonged to ST14, ST142, ST836 and ST1034 (one isolate each) (Table 2). Half of the isolates resistant to ciprofloxacin belonged to ST1524 (3/6 isolates); the others belonged to ST422 (belonging to CC3 as ST1524), ST124 and ST143 (Table 4). Isolates belonging to CC3 had a high level of resistance to ciprofloxacin, while the other two had a low level of resistance.

## 4. Discussion

In Italy, *H. influenzae* invasive disease is mainly caused by NTHi, while Hib persist as the most frequent of the encapsulated isolates, followed by Hif, as previously reported [23]. The predominance of NTHi isolates in invasive disease was documented elsewhere in countries with established anti-Hib vaccination, while the frequency of Hib is generally lower than that of Hif [5,6]. Antibiotic resistance is currently one of the most worrying problems in public health. In this study, we found that the principal antibiotic resistance characteristic of invasive *H. influenzae* isolates was resistance to ampicillin, which accounted for 21.7% of the isolates. This result is in line with other European studies, reporting similar ampicillin resistance levels in invasive isolates, with 24% in France during 2017 and 21.9% in Germany during 2016–2019 [17,20]. A lower prevalence was observed elsewhere, as in Portugal and Spain with 15.2 and 17.6% of the invasive isolates resistant to ampicillin, respectively [24,25]. Based on our previous studies, we found that ampicillin resistance has increased from 10.2% in the early 2000s to 24% during 2012–2016 [15,23]. This was due especially to the emergence of BLNAR isolates that now represent 9.4% of all the invasive isolates in Italy, carrying several PBP3 amino acid substitutions in the three functional motifs of the PBP3 and are responsible for ampicillin resistance [8,23]. β-lactamase producers had a TEM-1 type β-lactamase. ROB β-lactamase remained rare and was found in one case only, as reported already [8,23]. In a global survey of 2225 β-lactamase-positive strains, *H. influenzae* carrying β-lactamase harbored a TEM type β-lactamase in approximately 94% of cases, although with geographical variation, while ROB β-lactamase was less frequent, possibly due to a fitness cost of bearing the plasmid on which it is located [26,27]. According to substitutions in PBP3 protein, BLNAR isolates in our study showed the presence of several substitutions that have been classified as relevant for ampicillin resistance and were classified mainly as group IIb. This result was previously reported for BLNAR isolates in the literature, with A502V and N526K as the most frequent substitutions in BLNAR isolates [8,18].

Cefotaxime resistance accounted for 1.0% of invasive isolates, similar to 0.90% in invasive *H. influenzae* in Germany [20]. These isolates exhibited relevant PBP3 amino acid substitutions at the SSN motif (D350N and M377I) with other mutations including the S357N, S385T and R517H mutations. A previous study showed that the presence of R517H, N526K and S385T is associated with increased cefotaxime MICs [20].

Resistance to amoxicillin/clavulanate accounted for 4.3%, similar to 1.7% in Germany and 1.9% in the USA [20,28], and was prevalently associated with BLNAR with several substitutions in PBP3.

We identified invasive isolates resistant to ciprofloxacin in 1.5% of isolates for the first time in the last 25 years of surveillance. The emergence of resistance to fluoroquinolones in recent years was also observed in Spain where 4.4% of isolates were ciprofloxacin-resistant at a low level [25]. We found isolates resistant both at a low and high level, depending on the number of substitutions in GyrA and ParC. Ciprofloxacin-resistant NTHi isolates at a high level belonged to ST1524, as recently recovered in Ireland [29]. Isolates with low susceptibility belonging to ST422 have already been described in Norway and Japan [30,31]. The ST422 clone caused a regional outbreak in Japan and disseminated in the community [31]. In Norway, ST422 also showed β-lactam resistance, as in our study, where the clone was MDR. Meropenem retained high activity against *H. influenzae* invasive isolates representing a valid alternative for the treatment of invasive disease caused by resistant clones.

The high genetic diversity of resistant-NTHi isolates showed no predominant or regional clonal spreading in Italy, as demonstrated elsewhere [20,24,25]. Nevertheless, a third of isolates that produced β-lactamase belonged to ST103, a clone previously known as associated with β-lactamase production [23,24,25]. Moreover, one out of three BLNAR isolates belonged to ST12 or ST14, clones associated with β-lactam resistance by alteration in PBP3. The ST14 clonal group appears to be endemic in Scandinavia and caused an outbreak of respiratory tract infections in a nursing home in southern Sweden [32,33]. In our study, we did not observe outbreaks caused by ST14. This virulent clone was previously also reported in Canada in association with significant PBP3 mutations [34].

In conclusion, this study demonstrated that NTHi was the main cause of invasive disease due to *H. influenzae*, and its antibiotic resistance profile characterized the predominant isolates. Invasive *H. influenzae* isolates, with the exception of ampicillin, remained susceptible to clinically relevant antibiotics, with sporadic isolates resistant to amoxicillin/clavulanate, cefotaxime and ciprofloxacin. The detection of international resistant NTHi clones, such as ST103 and ST14, highlighted the importance of molecular surveillance for appropriate prevention and control strategies to counteract *H. influenzae* infection. Continuous monitoring and evaluation of antibiotic susceptibility, as well as genotyping, is crucial to identify promptly the emergence and diffusion of resistant clones.

## Figures and Tables

**Table 1:** Antibiotic susceptibility of 392 invasive *H. influenzae* isolates, Italy, 2017–2021.

	MIC (mg/L)			Susceptibility Category		Serotype		
Antibiotic Agent	MIC_50_	MIC_90_	Range	Susceptible	Resistant		Capsulated	
				No.; %	No.; %	NT-Hi (No. = 305)	Hib (No. = 49)	Non-Hib (No. = 38)
						Resistant Isolates		
Ampicillin	0.38	16	0.064–≥256	307; 78.3%	85; 21.7%	81; 26.6%	3; 6.1%	1; 2.6%
Amoxicillin/ clavulanate	0.50	2	0.008–12	374; 95.4%	17; 4.3%	17; 5.6%	0	0
Cefotaxime	0.023	0.064	0.006–1.5	388; 99.0%	4; 1.0%	4; 1.3%	0	0
Ciprofloxacin	0.008	0.016	0.002–≥32	386; 98.5%	6; 1.5%	5; 1.6%	0	1; 2.6%
Meropenem	0.064	0.19	0.012–0.38	392; 100%	0; 0.0%	0	0	0

**Table 2:** Deduced amino acid substitutions in the transpeptidase domain of PBP3 protein from 37 BLNAR, 2 BLPACR and 2 AMC-resistant isolates, Italy, 2017–2021.

Isolate	AM	AMC	ST	CC	*fts*I Allele	PBP3 Group	E347K	I348V	D350N	S357N	M377I	S385T	L389F	A437S	I449V	G490E	A502V	R517H	N526K	A530S	T532S	V547I	N569S
BLNAR																							
Hi685	2	0.5	14	3	209	I			*		*						*	*	*			*	*
Hi687	1.5	0.75	2027		137	m								*								*	*
Hi728	1.5	2	14	3	1	IIb			*		*						*		*			*	*
Hi729	2	1.5	1034	3	1	IIb			*		*						*		*			*	*
Hi733	2	8	12	12	2	IIb			*		*						*		*			*	*
Hi736	1.5	3	136	3	2	IIb			*		*						*		*			*	*
Hi754	1.5	2	1034	3	1	IIb			*		*						*		*			*	*
Hi756	1.5	1	1025	1021	21	IIc											T		*			*	*
Hi778	1.5	2	1412		37	IIa													*				
**Hi792**	2	2	142	142	26	III-like+	I		*	*	*	*	*					*			*	*	
Hi811	3	4	12	12	2	IIb			*		*						*		*			*	*
Hi825	1.5	4	12	12	227	m			*					*					*			*	*
Hi837	2	6	14	3	1	IIb			*		*						*		*			*	*
Hi879	1.5	2	107	107	43	m			*							*			*	*			
Hi883	1.5	1.5	1034	3	1	IIb			*		*						*		*			*	*
Hi885	1.5	2	1218	107	97	I											*	*					
Hi928	1.5	2	1390		2	IIb			*		*						*		*			*	*
Hi934	2	3	102	3	2	IIb			*		*						*		*			*	*
Hi937	1.5	2	14	3	1	IIb			*		*						*		*			*	*
Hi940	3	2	41	41	156	IIb											*		*			*	
Hi961	2	3	102	3	32	III-like			*	*	*	*						*			*	*	*
Hi974	2	3	145	11	24	IIc			*								T		*			*	*
Hi984	2	1.5	14	3	1	IIb			*		*						*		*			*	*
Hi985	1.5	2	396	396	5	IId									*				*			*	*
Hi995	1.5	2	425	425	38	IIc			*					*			T		*			*	*
Hi1016	2	1.5	12	12	33	III-like			*	*	*	*						*			*	*	*
Hi1029	2	4	947		24	IIc			*								T		*			*	*
Hi1030	1.5	1.5	34	34	71	m													*				
Hi1032	2	4	12	12	42	IIb										*	*		*			*	*
Hi1035	1.5	2	159	107	13	IIc			*								T		*			*	*
**Hi1047**	2	1.5	1034	3	1	IIb			*		*						*		*			*	*
Hi1058	1.5	1.5	107	107	43	m			*							*			*	*			
Hi1061	2	1.5	834	390	23	IIb			*		*					*	*		*			*	*
**Hi1074**	3	3	14	3	1	IIb			*		*						*		*				
Hi1086	1.5	4	949		17	IIb			*								*		*			*	
Hi1108	2	4	919		48	IIb											*		*				
Hi1109	1.5	2	57	57	113	IIc											T		*				
BLPACR																							
**Hi854**	256	8	836	836	33	III-like			*	*	*	*						*				*	
Hi1031	256	4	422	422	43	m			*							*			*				
AMCR																							
Hi691	1	12	2031	3	32	III-like			*	*	*	*						*			*	*	*
Hi744	1	3	136	3	2	IIb			*		*						*		*			*	*

Isolates marked in bold are resistant to cefotaxime; AM, ampicillin; AMC, amoxicillin/clavulanate; ST, sequence type; CC, clonal complex; BLNAR, β-lactamase-negative ampicillin-resistant; BLPACR, β-lactamase-positive amoxicillin/clavulanate-resistant; AMCR, amoxicillin/clavulanate- resistant; PBP3 groups are I, IIa, IIb, IIc, IId, III-like, III-like+, m, miscellaneous. An asterisk * indicates the presence of a substitution in the amino acid position indicated; I, substitution E347I; T, substitution A502T.

**Table 3:** Main characteristics of isolates resistant to ciprofloxacin.

Isolate	Year	Source	Serotype	ST	CC	MIC (mg/mL)						QRDR Substitutions	
						CIP	AM	AMC	CTX	MER	β-lac	GyrA	ParC
Hi805	2018	Blood	NTHi	1524	3	32	0.25	0.3	0.016	0.023	−	S84L−D88G	S84I
Hi900	2018	Blood	NTHi	143	3	32	0.25	0.2	0.016	0.094	−	S84L−D88Y	S84I
Hi914	2019	Blood	Hif	124	124	0.125	0.38	0.5	0.023	0.094	−	− D88N	−
Hi1010	2019	Blood	NTHi	1524	3	32	0.19	0.2	0.008	0.047	−	S84L−D88G	S84I
**Hi1031**	2020	CSF	NTHi	422	422	0.5	256	4	0.047	0.25	+	S84L−	S84R
Hi1062	2020	Blood	NTHi	1524	3	32	0.19	0.3	0.016	0.047	−	S84L−D88G	S84I

Isolate marked in bold is MDR; ST, sequence type; CC, clonal complex; AM, ampicillin; AMC, amoxicillin/clavulanate; CTX, cefotaxime; MER, meropenem; QRDR, quinolone resistance determining region; a minus sign indicates the absence of the corresponding substitution.

**Table 4:** Distribution of STs according to mechanism of ampicillin resistance in 85 isolates resistant to ampicillin.

β-Lactamase Producers		BLNAR	
ST	*n*	ST	*n*
6	3	12	5
34	1	14	6
57	1	34	1
69	1	41	1
103	15	57	1
155	1	102	2
160	3	107	2
165	1	136	1
180	1	142	1
184	1	145	1
388	4	159	1
422	1	396	1
513	1	425	1
644	1	834	1
716	1	919	1
760	1	947	1
786	1	949	1
836	4	1025	1
943	1	1034	4
946	1	1218	1
1667	1	1390	1
1850	2	1412	1
2129	1	2027	1
Total	48		37

BLNAR, β-lactamase-negative ampicillin-resistant.

## Data Availability

Sequence data were submitted to GenBank at NCBI under the BioProject PRJNA905936.

## Supplementary Materials

The following supporting information can be downloaded at: https://www.mdpi.com/article/10.3390/microorganisms11020315/s1, Table S1: List of Sequence type by serotype, grouped by clonal complex.
