# Supplementary material for: Antibiotic Susceptibility and Molecular Typing of Invasive Haemophilus influenzae Isolates, with Emergence of Ciprofloxacin Resistance, 2017–2021, Italy
Source: Microorganisms. 2023 Jan 26;11(2):315. doi: 10.3390/microorganisms11020315 (PMC9965257; doi:10.3390/microorganisms11020315)
Supplement: Supplementary file 1 [file microorganisms-11-00315-s001.zip › microorganisms-2144444-supplementary.pdf]

microorganisms

Article

Antibiotic Susceptibility and Molecular Typing of Invasive *Haemophilus influenzae* Isolates, with Emergence of Ciprofloxacin Resistance, 2017–2021, Italy

Maria Giufrè [1,*], Rita Cardines [1], Manuela Marra [2], Maria Carollo [2], Marina Cerquetti [1] and Paola Stefanelli [1]

[1] Department of Infectious Diseases, Istituto Superiore di Sanità, 00161 Rome, Italy
[2] Core Facilities Technical-Scientific Service (FAST), Istituto Superiore di Sanità, 00161 Rome, Italy
* Correspondence: maria.giufre@iss.it

Table S1. List of Sequence type by serotype, grouped by Clonal complex.

NTHi			Capsulated		
**CC (n)**	**ST**	**n**	**CC (n)**	**ST**	**n**
3 (20)	14	6	Hia		
	102	2	–	4	1
	136	2	23 (3)	23	2
	143	1		2053	1
	180	1	Hib		
	1034	4	6 (48)	6	38
	1524	3		78	1
	2031	1		95	1
11 (18)	103	15		101	1
	142	1		190	1
	145	1		227	3
	644	1		641	2
12 (6)	12	5		662	1
	786	1	222 (1)	116	1
34 (1)	34	2	Hie		
41 (1)	41	1	18 (3)	69	3
57 (1)	57	2	Hif		
107 (4)	107	2	105 (1)	105	1
	159	1	124 (30)	124	26
	1218	1		598	4
155 (1)	155	1			
165 (1)	165	1			
210 (1)	2129	1			
390 (1)	834	1			
393 (2)	1850	2			
395 (4)	388	4			
396 (1)	396	1			
422 (2)	422	1			
	1667	1			
425 (1)	425	1			
487 (3)	160	3			
513 (1)	513	1			
746 (1)	716	1			
836 (5)	836	5			

1025 (1)	1025	1	
−	184	1	
−	760	1	
−	919	1	
−	943	1	
−	946	1	
−	947	1	
−	949	1	
−	1390	1	
−	1412	1	
−	2027	1	
Total		88	87

CC, clonal complex; ST, sequence type.
